# Dental Pulp Stem Cell Recruitment Signals within Injured Dental Pulp Tissue

**DOI:** 10.3390/dj4020008

**Published:** 2016-03-25

**Authors:** Charlotte Rombouts, Charlotte Jeanneau, Athina Bakopoulou, Imad About

**Affiliations:** 1 Aix Marseille Université, CNRS, ISM UMR 7287, 13288, Marseille, France; romboutscharlotte@gmail.com (C.R.); charlotte.jeanneau@univ-amu.fr (C.J.); 2 Department of Fixed Prosthesis & Implant Prosthodontics, School of Dentistry, Aristotle University of Thessaloniki, GR-54124, Thessaloniki, Greece; abakopoulou@dent.auth.gr

**Keywords:** dental pulp stem cells, chemotaxis, migration assay

## Abstract

The recruitment of dental pulp stem cells (DPSC) is a prerequisite for the regeneration of dentin damaged by severe caries and/or mechanical injury. Understanding the complex process of DPSC recruitment will benefit future *in situ* tissue engineering applications based on the stimulation of endogenous DPSC for dentin pulp regeneration. The current known mobilization signals and subsequent migration of DPSC towards the lesion site, which is influenced by the pulp inflammatory state and the application of pulp capping materials, are reviewed. The research outcome of migration studies may be affected by the applied methodology, which should thus be chosen with care. Both the advantages and disadvantages of commonly used assays for investigating DPSC migration are discussed. This review highlights the fact that DPSC recruitment is dependent not only on the soluble chemotactic signals, but also on their interaction with neighboring cells and the extracellular matrix, which can be modified under pathological conditions. These are discussed to explain how these modifications lead to the stimulation of DPSC recruitment.

## 1. Introduction

The discovery of dental pulp stem cells (DPSC) [1] has opened up a whole new research field that offers many possibilities in dentistry. Indeed, DPSC can differentiate into odontoblast-like cells and produce reparative dentin [2]. This was demonstrated *in vivo* by seeding DPSC onto human dentin slices implanted into immunocompromised mice. These cells synthesized reparative dentin on the implanted dentin surface [3]. Although other localizations, such as peripheral nerve-associated glia [4], cannot be excluded, DPSC mainly reside in the perivascular regions of the pulp [5]. An elegant *ex vivo* tooth model with pulp injury has demonstrated increased proliferation of DPSC in the perivascular area, followed by migration to the lesion site [6]. These phenomena were not observed with dentin injuries, indicating that the DPSC activation and migration signals are only initiated upon pulp injury with a damaged odontoblast layer. This review will discuss the different cues for DPSC recruitment in injured dental pulp and the methodologies used to investigate DPSC migration. This knowledge can be used for *in situ* tissue engineering purposes that engage the tooth’s own cells in regenerating the lost or injured tissue. This is of particular interest in severe caries and cavity preparations with rotary instruments leading to pulp injury. In brief, bioactive molecules such as chemokines or growth factors encapsulated in scaffolds can be grafted into the lesion site where they will stimulate dentin pulp regeneration by enhancing the physiological activation and recruitment of endogenous DPSC. This approach offers several advantages over tissue engineering that involves the exogenous application of stem cells [7]. Indeed, the latter requires cell harvesting followed by *ex vivo* cultivation that is cost- and labor-intensive, and has a more complex clinical translation.

## 2. Migration of DPSC in Injured Pulp Microenvironment

### 2.1 Soluble Chemotactic Molecules

The migration of DPSC to the injured pulp site is a complex procedure that involves many processes which are not fully elucidated yet. To start with, DPSC need to be mobilized from their niche. In the literature, various DPSC mobilization signals have been reported which include growth factors (hepatocyte growth factor (HGF), basic fibroblast growth factor (FGF-2), transforming growth factor (TGFβ-1)), chemokines (monocyte chemoattractant protein-1 (MCP-1); chemokine (C-X-C motif) ligand 14 (CXCL14); stromal cell-derived factor 1 (SDF-1)), granulocyte-colony stimulating factor (G-CSF), sphingosine-1-phosphate (S1P), C5a and high mobility group box 1 (HMGB-1) [8,9,10,11,12,13,14]. These signals find their origin in different sources, including their release from the dentin matrix and their production by dental pulp cells. These sources are influenced by the local microenvironment defined by caries, the inflammatory state, mechanical injury and the application of restorative materials, as reviewed previously [15]. While these molecules are involved in DPSC recruitment, they can also be involved in other processes such as differentiation or inflammation (Table 1).

### 2.2. Both Chemotaxis and Haptotaxis are Required for DPSC Migration

Next, DPSC need to move through the extracellular matrix (ECM) towards the lesion site. The details of this process are not clear, but one can assume that both chemotaxis and haptotaxis are involved. Chemotaxis is guidance by a gradient of soluble chemical cues such as chemokines and cytokines, and also differing pH or reactive oxygen species levels [31]. For instance, in the case of carious insult, an acidic dissolution of the mineralized dentin matrix occurs, leading to the release of various bioactive molecules that are immobilized and sequestered within the matrix [32]. Chemotactic factors sequestered in the dentin include HGF [13], TGFβ-1 [9,33,34] and FGF-2 [9,17,35]. Dental pulp cells migrated *in vitro* in response to dentin ECM extracts [36], which was dependent on an active Rho pathway. Indeed, using an inhibitor of the Rho pathway, the migration-inducing effects of the dentin ECM extracts were abolished. Increased gene expression of pluripotency and mesenchymal markers was observed in the migrated pulp cell populations, suggesting that these migrated cell populations include DPSC. Enhanced dental pulp cell migration was also observed in response to dentin chips [37]. Haptotaxis is guidance by a gradient of immobilized ligands on the ECM such as collagens, fibronectin, laminins, matrix-bound chemoattractants, and/or adhesion molecules present on encountered cells [31]. For example, pulp ECM components, including laminin, have been shown to induce DPSC migration [9,36].

### 2.3. Extracellular Matrix Remodeling and DPSC Migration

Controlled remodeling of the ECM is also involved in cellular migration and is ensured by the action of different protease systems, including matrix metalloproteinases (MMPs). MMPs produced by odontoblasts [38] are sequestered in the dentin matrix, which are released upon dentin dissolution. Indeed, an enhanced presence of MMPs has been observed at carious lesion sites [39]. These host-derived MMPs, together with bacteria-derived MMPs and other enzymes, will further promote matrix breakdown and the release of sequestered bioactive molecules [40]. After pulp injury, matrix metalloproteinase-3 (MMP3) was shown to be highly increased, which stimulated endothelial cell migration [41]. In addition, MMPs have been shown to activate several bioactive molecules including TGFβ-1 [42]. Since MMPs not only breakdown the ECM [43], but also control chemokine activity and chemotactic gradients [44], it can be assumed that they are essential for interstitial migration of DPSC in the pulp tissue. Nevertheless, direct evidence of MMP-induced migration of DPSC is lacking and further research such as that on bone marrow-derived mesenchymal stem cells is needed [45,46].

### 2.4. SDF-1/CXCR4 Axis in Injured Pulp

A recently investigated migration cue in dental pulp is the SDF-1/CXCR4 axis. It is hypothesized that there is an elevated production of SDF-1 in injured pulp tissue, which will recruit proliferating dental pulp stem cells expressing the CXCR4 receptor where they will contribute to reparative dentin formation [26,27]. CXCR4 receptor was expressed intracellularly in STRO1^+^ (mesenchymal stem cell marker) dental pulp stem cells of the apical papilla (SCAP). Nevertheless, *in vitro* 2D and 3D migration assays have demonstrated increased migration of these SCAP in response to SDF-1. It is suggested that in response to a spatiotemporal variation of SDF-1 concentrations, the CXCR4 receptor is externalized. Indeed, immunofluorescence staining of migrating SCAP showed CXCR4 expression, whereas no expression was seen on non-migrating cells [47]. Also, for migration, cells will adopt a suitable morphology via intracellular cytoskeletal changes and focal adhesion formations. In response to SDF-1, focal adhesion proteins such as paxillin and focal adhesion kinase (FAK) were phosphorylated in DPSC. Stress fiber assembly, together with focal adhesion formation at the cell periphery, was observed [11].

## 3. Influence of Inflammation

Inflammation and tissue regeneration are tightly linked, and the dental pulp is no exception. Indeed, the microenvironment of the inflamed pulp differs from the healthy pulp and emits various signals required for regeneration to proceed. For instance, IL-1β, which is present in inflamed pulp, has been shown to induce MCP-1 secretion by dental pulp cells [48], which was shown to stimulate dental pulp regeneration [10]. Dental pulp cells challenged with lipopolysaccharide (LPS), a Gram negative bacterial wall component, increased their expression of DPSC chemotactic factors including SDF-1, CXCR4, MCP-1, FGF-2 and TGF-β1. This was associated with increased DPSC migration and the specific inhibition of the SDF-1/CXCR4 axis demonstrated its importance in this LPS-mediated migration [49]. Toll-like receptor (TLR) signaling, a major component of the innate immune system, is also involved in DPSC migration following bacterial insult [50,51]. Both Gram positive and Gram negative bacteria were able to increase Toll-like receptor 4 (TLR4) receptor expression in DPSC, which was associated with increased cytokine expression [51]. LPS-induced migration of murine dental papilla-derived odontoblast-like cells was also dependent on TLR4 receptor signaling [52].

The complement system is another major component of innate immunity [53]. It is a proteolytic cascade of more than 40 plasma and cellular proteins that are activated during inflammation with the aim of pathogen removal. The anaphylatoxins C3a and C5a are well-known chemoattractants of leukocytes towards the site of complement activation. Interestingly, these anaphylatoxins have also been studied in the context of tissue regeneration [54]. In particular, C5a has demonstrated the potential to stimulate pulp tissue regeneration. Indeed, C5a was produced by dental pulp fibroblasts challenged with LPS and lipoteichoic acid (LTA), a component of the Gram-positive bacterial wall [28,29]. C5a has been shown to recruit DPSC, which express the C5a receptor, to the dental pulp lesion site [8].

Another example of a molecule traditionally known for its role in inflammation and with discovered chemotactic potential is HMGB-1. It is secreted in inflammatory conditions by dendritic cells, macrophages and monocytes, and increases the production of inflammatory cytokines [14]. HMGB-1 expression was observed in inflamed pulp tissue, both in the nuclear and cytoplasmic regions of fibroblasts, inflammatory and vascular endothelial cells. A chemotactic effect of HMGB-1 was observed with a reorganization of F-actin that accumulated in the cell periphery of dental pulp cells. Accordingly, the receptor that mediates the chemotactic effect of HMGB-1 was upregulated as well in inflamed dental pulp tissues [14]. In addition, HMGB-1 has also been shown to promote the proliferation and odontoblastic differentiation of DPSC [55].

## 4. Influence of Pulp Capping Materials

**Figure 1:**
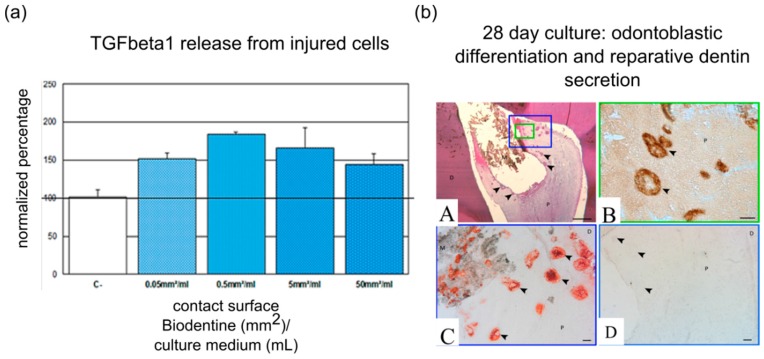
Biodentine and its effect on dental pulp regeneration. (**a**) Regardless of surface area, Biodentine induces TGFβ-1 release, a major chemoattractant for DPSC, from injured pulp cells; (**b**) Expression of odontoblast markers after pulp capping with Biodentine in entire tooth culture model. Numerous mineralization foci (arrowheads) can be viewed beneath the biomaterial after culture for two weeks and hematoxylin and eosin staining (**A**). Dentin sialoprotein (**B**) and nestin (**C**) are expressed in the mineralized foci, which represent an early form of reparative dentin (**D**). Abbreviations: D, dentin; P, pulp; M, biomaterial. Scale bars: A = 500 µm; B,C,D = 100 µm. Modified with permission from [18].

Several pulp capping materials, such as calcium silicate-based cements, have been shown to positively influence the dentin-pulp regenerative process [18,56]. This is mainly due to the release of Ca^2+^ ions, which are known to stimulate hard tissue formation [57,58,59]. The release of silicon ions from calcium silicate-based cements also induced odontoblastic differentiation of DPSC. It is suggested that silicon ions act through calcium channels and mitogen-activated protein kinase/extracellular signal-regulated kinases (MAPK/ERK) signaling [60,61]. Besides the established odontoblastic differentiation of DPSC, dental materials can also stimulate cellular migration. For instance, mineral trioxide aggregate (MTA) increased the early and short-term migration of SCAP [62]. BioAggregate and MTA increased DPSC migration in an *in vitro* scratch assay [63]. The bioceramic putty iRoot BP Plus induced dental pulp cell migration, which was associated with focal adhesion formation and stress fiber assembly [64]. Biodentine also induced DPSC migration, which was associated with an increased chemokine expression, such as CXCR4, MCP-1 and SDF-1 [65]. The released calcium can be held partially responsible for the increased cellular migration, as was shown for bone marrow-derived stem cells [66]. Besides this calcium release, calcium hydroxide and MTA were able to mobilize bioactive molecules sequestered in the dentin matrix [33,67], which can contribute to their migration-stimulating effects. Furthermore, Biodentine induced TGFβ-1 secretion by dental pulp fibroblasts [18], which has been shown to attract DPSC [16] (Figure 1). A recent study has demonstrated that fluoride-based restorative materials impact DPSC migration [68]. Indeed, the long-term release of low fluoride levels increased the migratory response of DPSC to the chemotactic factors SDF-1 and TGFβ-1.

On the other hand, several materials demonstrated anti-migratory effects. For example, resin-based materials containing 2-hydroxyethyl methacrylate (HEMA) diminished DPSC migration [69]. HEMA has also been shown to decrease FGF-2 secretion by dental pulp cells [70], which may (partially) explain the decreased DPSC migration. An interesting study points out the importance of the local microenvironment, such as nutrient deprivation, in the pulp response to resin-based materials [71]. A concentration-dependent decrease in DPSC proliferation and migration was observed when exposed to TEGDMA (triethylene-glycol-dimethacrylate). Mineralization was also delayed when exposed to sub-toxic TEGDMA concentrations. A conditioned medium obtained from serum-deprived dental pulp cell cultures was able to attenuate these deleterious effects of sub-toxic TEGDMA concentrations, except for DPSC migration, which was attributed to increased FGF-2 and TGFβ-1 concentrations.

## 5. Methodologies Used to Study Dental Pulp Stem Cell Recruitment

### 5.1. *In vitro* Boyden Chamber (Transwell Assay)

**Figure 2:**
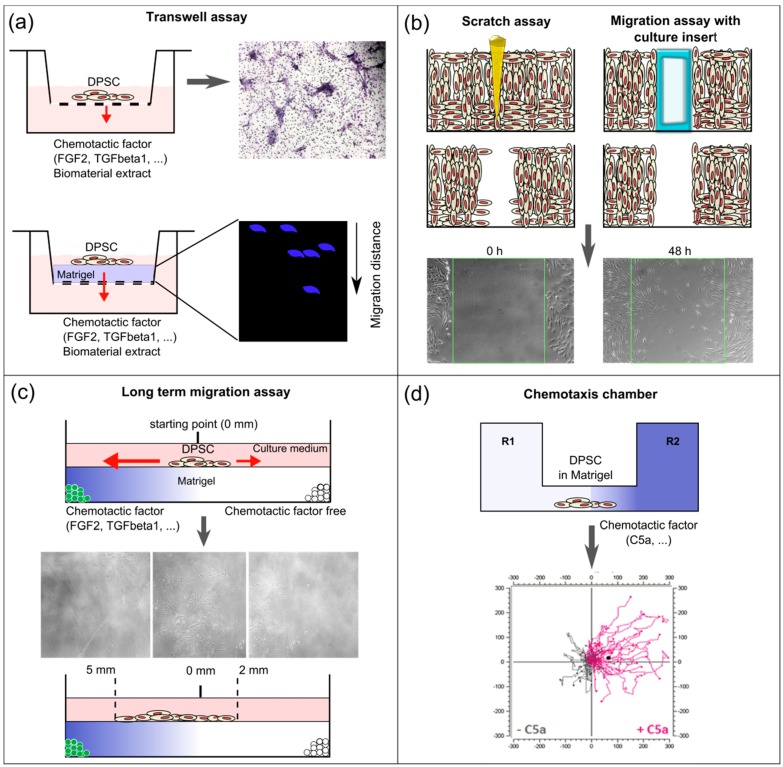
Schematic overview of the different *in vitro* assays used to study DPSC recruitment and migration. (**a**) On the upper panel, a classical Trans-well assay used to evaluate the chemotactic potential of bioactive molecules and biomaterials (extracts), with cells seeded on the upper side of the membrane. Results are obtained by swabbing the non-migrated cells from the upper side of the membrane followed by histological staining of the migrated cells on the lower side of the membrane. These can be counted and quantified. On the lower panel, a modified Trans-well assay where the porous membrane is coated with an extracellular matrix (ECM)-like gel (e.g., Matrigel, collagen), on which cells are seeded. Gels can be retrieved to evaluate the migrated distance of cells (e.g., by cell nuclear staining using 4’,6-diamidino-2-phenylindole (DAPI) (blue) and confocal microscopy); (**b**) On the left, scratch assay to evaluate cellular migration. A scratch is made (usually using a pipette point) on confluent cell cultures. On the right, a modified assay to study cellular migration is represented. A culture insert is placed, around which cells are cultured. When the culture is confluent, the insert is removed leaving behind a reproducible cell-free zone with sharp borders, as opposed to the scratch assay. With both techniques, cellular migration into the cell-free zone is followed up microscopically and can be quantified using specialized software. A chemotactic gradient is absent in these assays; (**c**) Culture model to study long-term migration following the controlled release of chemotactic factors. Poly(lactic-co-glycolic acid) (PLGA) microspheres with or without chemotactic factors are embedded in Matrigel in a graduated tissue culture chamber. DPSC are seeded in the middle and their migration can be followed microscopically over long time periods (up to 10 days); (**d**) Specialized *in vitro* chemotaxis chamber. A stable chemotactic gradient is established by injecting a solution of chemotactic factor in one of the reservoirs. Cells are seeded in the observation area containing Matrigel in the middle of the chamber. Real-time monitoring of cellular migration can be performed up to 48 h. Quantitative evaluation is carried out by specialized software. Individual cells are tracked and the center of mass (CAM) is calculated. Trajectory plots can be presented graphically.

The most commonly used chemotaxis assays are based on the Boyden chamber and are often referred to as Trans-well assays [72,73] (Figure 2a). In these assays, an insert with a porous membrane is used, with cells seeded on the upper site, which is placed into a culture well containing a medium with chemotactic factors. After a specific culture period, the cells on the upper site are swabbed away, leaving behind the cells that actively transmigrated to the other site of the membrane. The reproducibility and facility of this assay, together with its cost effectiveness, has made it a widely used tool to evaluate chemotactic potential of molecules. For instance, the chemotactic effect of SDF-1, S1P, EGF, FGF-2 and TGFβ-1 on DPSC has been demonstrated using a Trans-well assay [9,11]. The chemotactic potential of pulp and dentin ECM matrix extracts has been shown as well [36]. Haptotactic effects can be evaluated with a specific setting, as demonstrated for the so-called SIBLING molecules (small integrin-binding ligand, N-linked glycoproteins) [74]: dentin matrix protein-1 (DMP1), bone sialoprotein (BSP), and osteopontin (OPN). To this end, the porous membranes were coated with SIBLING solutions on the lower side, after which cells were seeded on the upper side. Haptotactic migration was observed with all three SIBLING proteins, which was dependent on the interaction between cellular αVβ3 integrin and the highly conserved integrin-binding tripeptide, RGD, on the protein.

Various modifications of the Trans-well assay have been introduced over the past years. For example, the porous membrane can be coated with a gel that simulates the ECM, most often a collagen-based gel or Matrigel. In this way, one can get a step closer to the *in vivo* situation without too much effort. Indeed, chemotaxis *in vivo* is not only determined by the presence of a chemotactic gradient, but also by interaction between chemokines and the surrounding matrix and cellular interactions. In particular, the so-called mechanical tissue properties, stiffness and porosity, as well as chemokines, will together define the cell behavior, including intracellular signaling cytoskeletal changes, and integrin-focal interactions [75]. Adding a 3D-structure that cells need to migrate through requires cells to modify their shape and interact with the matrix, which forms both a barrier and a substrate [76]. Collagen is the most commonly used substrate for 3D migration assays. This is most likely because pulp ECM consists predominantly of collagen type I and III fibrils [77]. Suzuki and coauthors have used a modified Trans-well insert migration assay with 3D collagen to study the chemotactic potential of FGF-2, SDF-1 and bone morphogenetic protein 7 (BMP7) over a longer time period. DPSC were seeded onto the collagen gel in a Trans-well insert that was placed in culture medium with the respective molecules. After seven days, the collagen gels were retrieved, fixed and 4’,6-diamidino-2-phenylindole (DAPI) stained for further analysis with confocal microscopy [17].

Several disadvantages associated with the Trans-well assay should be noted. Firstly, chemotactic gradients are diffusion-driven and thus rather unstable [7]. Secondly, this methodology does not allow for a real-time follow-up of the migration process. To respond to these limitations, *in vitro* chemotaxis chambers have been developed that allow for real-time monitoring of cellular migration [78] (Figure 2d). They are the size of a microscopic slide and have two reservoirs that are connected by an interstitial observational area where cells are seeded in Matrigel. These cells are subjected to a chemotactic gradient via a concentration-distribution of the solution injected into one of the reservoirs. With this approach, a more stable chemotactic gradient is established, and cells can be tracked for 48 h and over a longer distance with real-time microscopic imaging. This technique has been successfully applied to study the chemotactic potential of C5a on DPSC [8]. By calculating the displacement of the center of mass (CAM) of STRO1^+^ cells over 48 h, a clear migration following the C5a gradient was shown. Another culture model to study cellular migration that has been developed is based on the use of graduated tissue culture chambers coated with Matrigel (Figure 2c). In this model, cells are seeded in a droplet in the middle. Microspheres with different molecules are embedded on opposite sides in the culture chamber and cellular migration can be followed microscopically. In this way, longer migration periods (up to 10 days) in response to the controlled release of FGF-2 and TGFβ-1 from microspheres have been studied in real-time [16].

### 5.2. *In vitro* Scratch Assay

Another cost-effective technique to study cell migration is the scratch assay, also referred to as the wound healing assay. A scratch is made in a confluent cell layer followed through the evaluation of cellular migration to close the cell-free gap. A modified version consists of growing cells around a culture insert which is removed when cells reach confluence, leaving behind a more reproducible gap with sharp edges (Figure 2b). This methodology does not allow the study of chemotaxis as a chemotactic gradient cannot be established. Nevertheless, it can give insight into the impact of different molecules or materials on cellular migration. This assay has mainly been used to study the influence of materials such as Biodentine, BioAggregate, ProRoot MTA and HEMA on DPSC migration [63,65,69].

### 5.3. Cell Migration Tracking *in vivo*

*In vivo* migration assays typically consist of tracking labeled transplanted cells into animal models by magnetic resonance imaging, optical fluorescence imaging, positron emission tomography and single-photon emission computed tomography [7]. These do not allow, however, for the assessment of endogenous cell recruitment, and require rather complex and expensive experimental procedures. End-point *in vivo* assays, on the other hand, can be used to study endogenous cell recruitment. These are based on the immunohistological identification of proliferating cells at different time points, following which the cellular migration route can be reconstructed. For example, a rat model was used to evaluate the spatio-temporal evolution of proliferating pulp cells in response to pulp injury treated with agarose beads containing bioactive ECM components [79]. Rats were sacrificed after 1, 3, 8, 15 and 30 days, and pulp tissues were immunostained using proliferating cell nuclear antigen (PCNA). Based on their findings, it could be deduced that cells migrated from the central part of the radicular pulp to the sub-odontoblast cell layer and then from the apical root to the crown. *Ex vivo* assays, based on the same approach, form a suitable alternative to study *in vivo* endogenous stem cell recruitment.

### 5.4. *Ex vivo* Entire Tooth Culture Reproduces in vivo Migration Conditions

**Figure 3:**
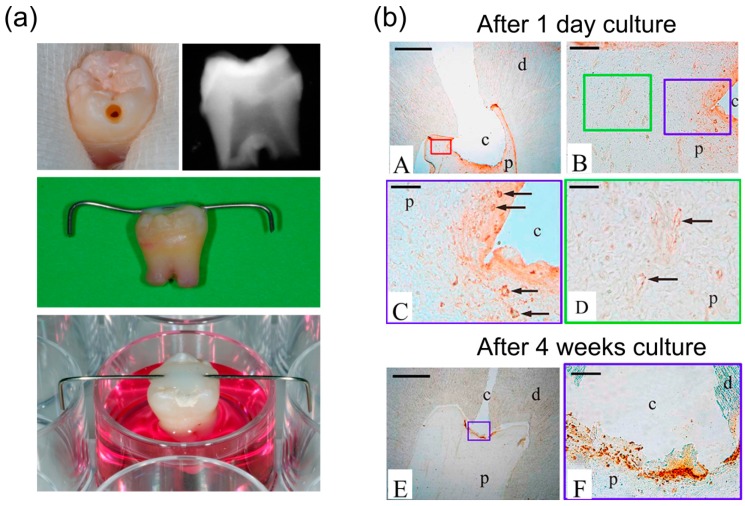
Overview of the *ex vivo* tooth model and its application for studying DPSC migration. (**a**) The pulp cavity was prepared in a clean immature third molar. X-ray imaging showed that the cavity is reaching the pulp. After cavity preparation, teeth were suspended with their root canals in culture medium with the aid of a steel wire fixed on the crown; (**b**) Representative histological images of teeth cultured with medium containing 5-bromo-20-deoxyuridine (BrdU) during one day after cavity preparation, followed with medium without BrdU for four weeks. After one day, the labelling was localized in the nuclei of cells in the perivascular area. The BrdU immunolabelling exhibited a gradient. It was strong in the blood vessels surrounding the cavity (**B** and **C**) and decreased in those away from the cavity (**B**, **D**). At four weeks, the immunolabelling was localized at the cavity area only (**E** and **F**). *Abbreviations*: c: cavity; d: dentin; p: pulp; arrows indicate vessels. Scale bars in (**A**, **E**) 1 mm; (**B**, **F**) 100 µm; (**C** and **D**) 50 µm. Modified with permission from [6].

For instance, an *ex vivo* culture model of entire human teeth has been developed [6] (Figure 3). Immature third molars extracted for orthodontic reasons are cleaned, and pulp injuries are made with sterilized instruments, after which they are put in culture medium as illustrated in Figure 3. This model has been used to investigate the migration of DPSC in response to pulp injury. To this end, teeth with a pulp cavity were cultured in medium containing 5-bromo-20-deoxyuridine (BrdU) for one day after pulp injury, after which teeth were cultured in culture medium without BrdU. In this way, it could be demonstrated that directly after pulp injury, proliferating cells were at the perivascular area, whereas after two weeks of culture these proliferating cells were detected in the vicinity of the cavity. This model is a cost-effective alternative to *in vivo* migration studies. Given that this is an entire tooth culture, it simulates the interaction of migrating cells with ECM components, reproduces interactions between the different pulp cell populations and the production of chemotactic factors by these cells. Nevertheless, some disadvantages can be noted, including the absence of a functional vascular system in the pulp and the limited simulation of all inflammatory reactions. Histological processing is also cumbersome, like for the above-mentioned *in vivo* assays.

## 6. Potential Use in Tissue Engineering

Three major processes can be discerned in tissue regeneration: the recruitment of stem/progenitor cells to the lesion site, the differentiation of these cells and the maturation of the newly formed tissue [80]. Tissue engineering approaches are aimed to optimize these processes. Here, we focus on the modulation of endogenous DPSC recruitment to the pulp lesion site, which can be categorized as *in situ* tissue engineering [20]. Specific chemotactic factors can be used to alter the host microenvironment and stimulate as such the migration of progenitor cells towards the lesion site [20]. Given that the majority of these soluble chemotactic factors have a rather short half-life and are expensive [81], appropriate delivery vehicles are required. Biodegradable microspheres are extensively investigated as they have promising properties including a controlled release of the bioactive molecule and ease of preparation, often with ground substances that are already clinically approved by the US Food and Drug Administration (e.g., poly(lactic-co-glycolic acid) (PLGA)) [82].

The SDF-1/CXCR4 migratory axis is being explored by various researchers for cell homing purposes. For example, poly-L-lactide scaffolds containing SDF-1, which were implanted between the skin and deep muscle of mice, induced stem cell recruitment [83]. Other chemotactic factors that have been investigated include FGF-2 and TGFβ-1, which are encapsulated in biodegradable PLGA microspheres [16]. It was observed that these growth factor-containing microspheres stimulate dental pulp cell proliferation and migration. An *in vivo* study has demonstrated the benefit of a composite material consisting of PLGA microspheres loaded with TGFβ-1 that have been incorporated in calcium phosphate cement [84]. This composite was used for pulp capping in goat incisors, and the subsequent histological evaluation showed that the composite with the highest TGFβ-1 concentration was able to stimulate odontoblastic differentiation of DPSC and associated tertiary dentin formation.

Besides exogenous application of chemotactic factors, other elements in pulp injury treatments can stimulate endogenous DPSC recruitment. For example, several pulp capping materials already used in the clinic will positively influence the dentin pulp regeneration process, partly by enhancing DPSC migration towards the lesion site. Interestingly, a recent study has showed a stimulating effect of magnetic nanocomposite scaffolds made of magnetite nanoparticles and polycaprolactone on DPSC migration [85]. Ethylenediaminetetraacetic acid (EDTA) preconditioning of dentin has also been demonstrated to increase DPSC migration [86], which can be attributed to the release of sequestered chemotactic factors in the dentin matrix.

In the clinic, DPSC recruitment to treat pulp injury will most likely include the application of a biodegradable device containing chemotactic factors overlaid with a bioactive pulp capping material.

## 7. Conclusions

Stem cell mobilization and recruitment appears as a complex process. It requires both soluble chemotactic factors and insoluble extracellular matrix and environmental factors, which constitute the pulp microenvironment. While some of these soluble factors are sequestered in the dentin, others originate from plasma or can be synthesized by pulp fibroblasts. Insoluble factors usually surround the stem cells and are necessary for the guided migration of these cells. Pathological conditions with moderate inflammation following pulp injury and after applying direct pulp capping bioactive materials lead to a modification of the pulp microenvironment. This modified microenvironment will promote the dental pulp stem cell recruitment that is required to replace the missing odontoblasts and to synthesize the pulp protective reparative dentin.

Knowledge of stem cell mobilization and migration signals from a variety of *in vitro*, *in vivo* and *ex vivo* methodologies show that each has specific advantages and disadvantages. While *in vitro* assays allow for a rapid and cost-effective evaluation of these signals, they should be considered as a first research step. Both *ex vivo* and *in vivo* assays will provide a more thorough understanding of stem cell mobilization and migration in the pulp.

These methodologies also serve the field of *in situ* tissue engineering that targets the tooth’s own stem cells to stimulate dentin pulp regeneration. The explored treatment approaches include the controlled delivery of chemotactic factors from biodegradable devices such as microspheres. The ultimate goal of this procedure is to obtain reparative dentin formation by DPSC after their differentiation into odontoblast-like cells leading to dentin pulp regeneration, while preserving the pulp volume.

## Figures and Tables

**Table 1:** Overview of migration signals for dental pulp stem cells (DPSC) with their corresponding receptors, and their specific roles in dentin pulp regeneration and inflammation.

Chemotactic Molecule	Receptor	Inflammation	Regeneration	Role	Reference
HGF	c-Met		x	Recruitment of DPSC	[13]
FGF-2	FGF receptors 1 and 2		x	Recruitment of DPSC and pulp cell proliferation	[9,16,17]
TGFβ-1	TGF-β1 receptors I and II		x	Recruitment of DPSC and odontoblastic differentiation	[9,16,18,19]
MCP-1	CCR2		x	Recruitment of DPSC and stem cell homing*	[10,20]
CXCL14	C-X-C chemokine receptor type 4 (CXCR4)	x	x	Recruitment of DPSC and mediator of immune cell migration*	[10,21]
G-CSF	G-CSF receptor	x	x	DPSC mobilization and anti-inflammatory properties	[12,22,23]
S1P	S1P receptor 1–3	x	x	Pleiotropic actions including recruitment of DPSC and inflammatory effects*	[9,24,25]
SDF-1	CXCR4		x	Recruitment of DPSC and stem cell homing*	[11,20,26,27]
C5a	C5a receptor	x	x	Recruitment of DPCS and inflammatory cells*	[8,28,29]
HMGB-1	receptor for advanced glycation end products (RAGE)	x	x	Recruitment of DPCS and production of inflammatory cytokines*	[14,30]

* Demonstrated outside dental pulp context.

## Abbreviations

The following abbreviations are used in this manuscript:

BMP: bone morphogenetic protein
BrdU: 5-bromo-20-deoxyuridine
BSP: bone sialoprotein
CAM: center of mass
CXCL14: chemokine (C-X-C motif) ligand 14
CXCR4: C-X-C chemokine receptor type 4
DMP-1: dentin matrix protein-1
DPSC: dental pulp stem cells
DAPI: 4’,6-diamidino-2-phenylindole
ECM: extracellular matrix
EDTA: ethylenediaminetetraacetic acid
FAK: focal adhesion kinase
FGF-2: basic fibroblast growth factor
G-CSF: granulocyte-colony stimulating factor
HEMA: 2-hydroxyethyl methacrylate
HGF: hepatocyte growth factor
HMGB-1: high mobility group box 1
LPS: lipopolysaccharide
LTA: lipoteichoic acid
MAPK/ERK: mitogen-activated protein kinase/extracellular signal-regulated kinases
MCP-1: monocyte chemoattractant protein 1
MMPs: matrix metalloproteinases
MMP3: matrix metalloproteinase-3
MTA: mineral trioxide aggregate
OPN: osteopontin
PLGA: poly(lactic-co-glycolic acid)
RAGE: receptor for advanced glycation end products
S1P: sphingosine-1-phosphatase
SCAP: dental pulp stem cells of the apical papilla
SDF-1: stromal cell-derived factor 1
SIBLING: small integrin-binding ligand, N-linked glycoproteins
TEGDMA: triethylene-glycol- dimethacrylate
TGFβ-1: transforming growth factor β 1
TLR: Toll-like receptor
TLR4: Toll-like receptor 4
